# Dhole pack size variation: Assessing the effect of Prey availability and Apex predator

**DOI:** 10.1002/ece3.7380

**Published:** 2021-03-23

**Authors:** Aishwarya Bhandari, Pallavi Ghaskadbi, Parag Nigam, Bilal Habib

**Affiliations:** ^1^ Department of Animal Ecology and Conservation Biology Wildlife Institute of India Dehradun India; ^2^ Department of Wildlife Health and Management Wildlife Institute of India Dehradun India

**Keywords:** Apex predator, *Cuon alpinus*, dhole, intraguild interactions, pack size

## Abstract

In multipredator systems, group sizes of social carnivores are shaped by the asymmetric intraguild interactions. Subordinate social carnivores experience low recruitment rates as an outcome of predation pressure. In South and Southeast Asia, the Tiger (*Panthera tigris*), Dhole (*Cuon alpinus*), and Leopard (*Panthera pardus*) form a widely distributed sympatric guild of large carnivores, wherein tigers are the apex predators followed by dhole and leopard. In this study, we attempted to understand the variation in pack size of a social carnivore, the dhole, at two neighboring sites in the Central Indian landscape. We further evaluated local‐scale patterns of variation in pack size at a larger scale by doing a distribution‐wide assessment across the dhole ranging countries. At the local scale, we found an inverse relationship between the density of tiger and pack size of dhole while accounting for variability in resources and habitat heterogeneity. Larger dhole packs (16.8 ± 3.1) were observed at the site where the tiger density was low (0.46/100 km^2^), whereas a smaller pack size (6.4 ± 1.3) was observed in the site with high tiger density (5.36/100 km^2^). Our results for the distribution‐wide assessment were concordant with local‐scale results, showing a negative association of pack size with the tiger densities (effect size −0.77) and a positive association with the prey abundance (effect size 0.64). The study advances our understanding to answer the age‐old question of “what drives the pack size of social predators in a multipredator system?” This study also highlights the importance of understanding demographic responses of subordinate predator for varying competitor densities, often helpful in making informed decisions for conservation and management strategies such as population recovery and translocation of species.

## Graphical abstract

This study focuses on understanding responses of a subordinate but endangered canid, the dhole, to varying densities of tigers. We found an inverse pattern between the apex predator density and group size of a subordinate predator while accounting for variability in resources and habitat heterogeneity. Our study highlights the importance of understanding species responses to each other, crucial for effective conservation and management implications.

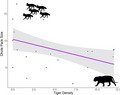

## 1 INTRODUCTION

In the past few decades, the focus of wildlife biology studies has shifted from single species targeted approach to an ecosystem conservation approach (Linnell & Strand, 2000). This holistic approach unveils how interspecific interactions can alter community structures and ecosystem functioning (Ford & Goheen, 2015). Often considered as keystone species, large carnivores regulate the ecosystem functioning through top‐down mechanisms (Caro & O'Doherty, 1999; Ritchie & Johnson, 2009). One such mechanism is intraguild competition that shapes the predatory guild (Palomares & Caro, 1999). However, in a multipredator system, the strength and direction of competition are complex. Therefore, it is imperative to understand behavioral and demographic responses of carnivores to each other, for safeguarding their viable populations and for maintaining ecosystem equilibrium.

One such multipredator system found in South and Southeast Asian forests is of the tiger, dhole, and leopard. Wherein, the two big cats are solitary and the dhole is a social canid. Based on their physiological demands and competitive abilities, tigers (180–245 kg) are considered to be top predators followed by leopards (46–65 kg) and dholes (10–21 kg) (Steinmetz et al., 2013). In the Indian subcontinent, dholes have been widely studied along with tigers and leopards under various ecological settings to understand the dynamics of intraguild competition. The findings suggest that the coexistence among these carnivores is facilitated by abundant prey resources (Acharya, 2007; Johnsingh, 1992; Karanth & Sunquist, 1995, 2000; Wang & Macdonald, 2009; Wegge et al., 2009). However, when resources are scarce, species might show fine‐scale adjustments in spatiotemporal activity patterns to allocate resources and to avoid competition from the dominant carnivore (Karanth et al., 2017; Rayan & Linkie, 2016). For example, a study conducted in Kuiburi National Park, Thailand; found tiger presence to be solely correlated with prey rich sites, whereas, presence probability of dhole and leopard was a trade‐off between prey availability and active spatial avoidance of tigers (Steinmetz et al., 2013). Whereas, tiger depleted system of Northern Laos has shown a significant increase in site occupancy of dholes, (Rasphone et al., 2019).

However, a crucial aspect that requires further understanding is that of the social structure dholes live in. In social carnivores, group size is a vital survival strategy and has definite fitness consequences (Courchamp & Macdonald, 2001; Stephens & Sutherland, 1999). For example, the pack size of African wild dog (*Lycon pictus*), may vary from 3 to 20 individuals (Creel & Creel, 1995); however, the optimum pack size of at least 4–5 individuals is crucial for foraging, breeding, and survival (Courchamp et al., 2000). Similarly, in African lions (*Panthera leo leo*), social group size may vary between 2–35, depending on social factors like social stress, kinship, alloparental care, and site‐specific environmental factors like resource distribution and availability (Loveridge et al., 2009; Macdonald, 1983; Orsdol et al., 1985).

Multiple ecological correlations can explain the rate at which conspecifics interact with each other and form social units. Prey abundance, composition, and distribution are known to influence social structuring in carnivores (Périquet et al., 2015). For instance, under scarce prey conditions in the African savanna, lions either become solitary or form large groups (to kill large‐bodied prey) to maximize their food intake (Mbizah et al., 2020). Carnivore group sizes might also vary in response to landscape fragmentation, Atwood (2006) recorded coyote (*Canis latrans*) group sizes to be large in aggregated forest patches in comparison with fragmented and corridor patches. Similarly, intraspecific group size variation in carnivores can potentially be an outcome of seasonality, disease prevalence, and anthropogenic disturbances (Gusset & Macdonald, 2010).

Asymmetric intraguild interactions are also known to shape group sizes in social carnivores. Maintaining larger groups would be advantageous for foraging, breeding, and coexisting with larger predators (Courchamp & Macdonald, 2001). However, in the African savanna ecosystem, the group sizes of subordinate predators such as African wild dogs, and spotted hyenas (*Crocuta crocuta*) (Creel & Creel, 1996, 1998; M'soka et al., 2016; Périquet et al., 2015) have been recorded to be inversely related to lion densities. The reduced group sizes in subordinate competitors are an outcome of predation pressure, low recruitment rates, and reduced energy gains due to the inability to guard kills against apex predators (Courchamp & Macdonald, 2001).

According to the International Union for Conservation of Nature, India has the largest dhole population across the dhole‐ranging countries (Kamler, 2015). Interestingly, within India there is an apparent variation in dhole pack sizes; the smallest dhole packs of 2–3 individuals are reported from the evergreen forest and rugged terrain of northeast, whereas, the average pack size of around 12 to 14 individuals is reported from the dry deciduous forest of Central and Southern India (Bashir et al., 2014; Majumder et al., 2011; Ramesh et al., 2012; Selvan et al., 2014). In comparison with India, the dhole population in Southeast Asia is much more fragmented, and reported pack sizes are also small (~ 4 to 5) (Kamler et al., 2020). The smaller pack sizes in Southeast Asia are hypothesized to be an adaptation to stalk and coordinate hunt through the thick rainforests (Kawanishi & Sunquist, 2008). Kawanishi and Sunquist (2008) also suggested smaller packs to be energetically beneficial due to the scarcity of large‐bodied prey. However, multiple diet studies show site‐specific prey preference by dholes, ranging from small to large body‐sized prey (Grassman et al., 2005, Slangsingha et al., 2007, Charaspet et al. 2015; Khoewsree et al., 2020). Additionally, a few studies suggest, rather than prey size class, it is the prey availability and its spatial distribution that potentially impacts prey choice and pack size (Hayward et al., 2014; Kamler et al. 2020). So far, most of the competing hypotheses for dhole pack size variation are only based on diet studies. However, crucial ecological correlates such as varying competitor density (Green et al., 2019), prey availability (Macdonald, 1983), habitat contiguity (Atwood, 2006), and terrain complexity (Kamler et al. 2020), have been missing from the previous studies.

We observed a significant variation in pack size of dholes at the two neighboring protected areas having similar ecological settings, Tadoba Andhari Tiger Reserve and Navegaon Nagzira Tiger Reserve in the Central Indian Landscape, Maharashtra, India. To understand this intraspecific variation, we investigated underlying factors that potentially govern group size variation in social carnivores. We further elucidate our site‐specific patterns at a larger scale by doing a distribution‐wide assessment of pack size across dhole range countries. Based on previous knowledge we attempted to answer: 1) How does intraguild competition with tiger's effect dhole pack size? Asymmetric intraguild competition is a crucial aspect that shapes carnivore group size (Macdonald, 1983), high intraguild competition from larger predators is known to result in reduced group sizes and low recruitment rates in subordinate social carnivores (Groom et al., 2017). Therefore, we hypothesize that dhole packs would be smaller in high tiger density areas. 2) How prey availability correlates to dhole pack size? Variation in carnivore group size is a demographic adaptation to varying prey availability (Périquet et al., 2015). Therefore, we hypothesize that dhole pack size would be positively correlated to higher prey density, whereas it would be physiologically beneficial to be in small groups when prey resources are scarce. 3) Intraspecific group size variation is also known to be a function of habitat contiguity (Atwood, 2006), therefore, we hypothesize that larger dhole packs would positively correlate to contiguous undisturbed habitat patches, whereas small packs would be associated with small and disturbed habitat patches. 4) Dhole packs have been observed to be smaller in hilly terrain in comparison with flat terrains as an adaptation to the spatial distribution of prey and its availability (Kamler et al. 2020), therefore, we also investigated if pack size is influenced by terrain type.

## 2 METHODS AND MATERIALS

### 2.1 Study area

**FIGURE 1:**
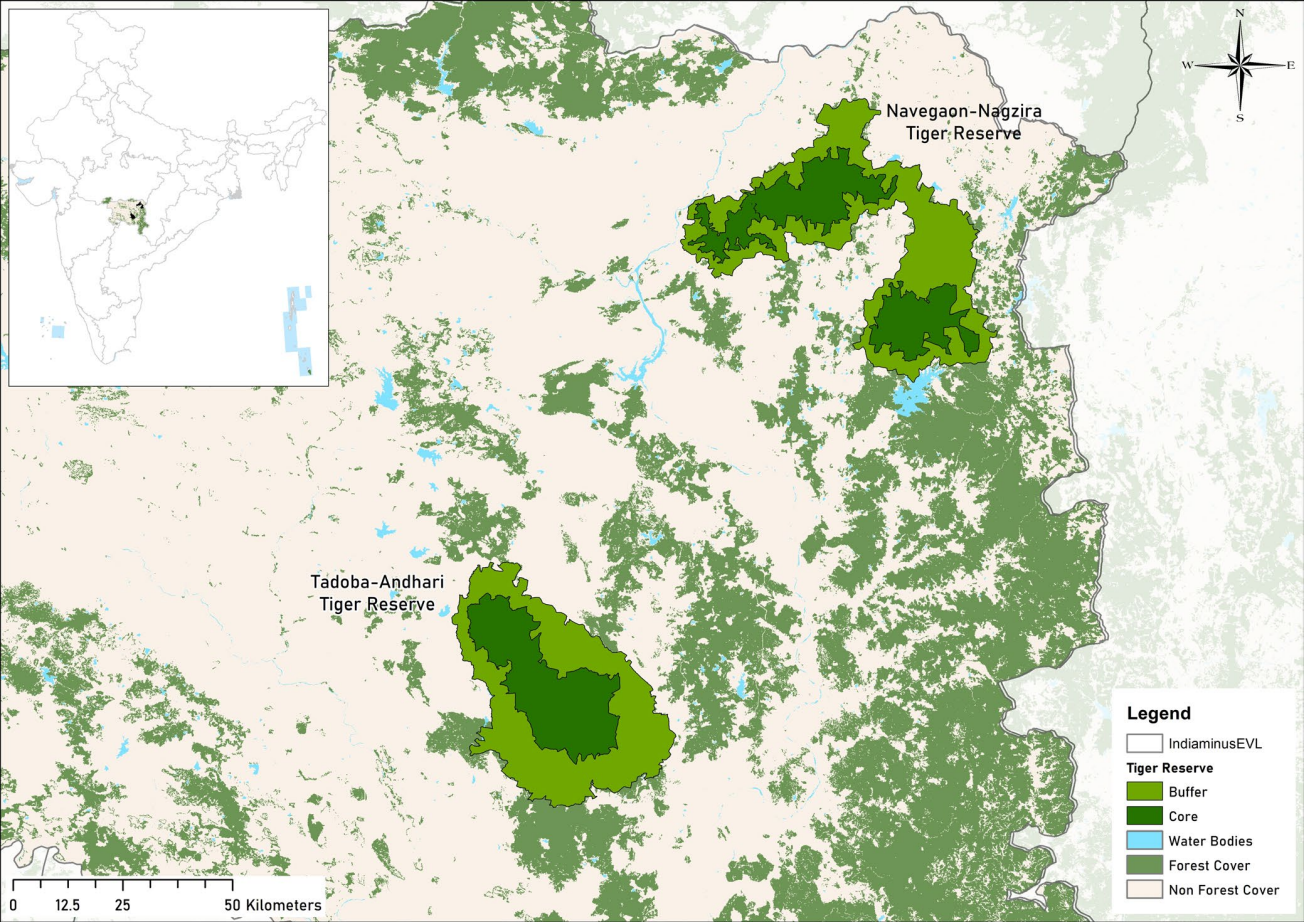
Map showing Tadoba Andhari Tiger Reserve and Navegaon Nagzira Tiger Reserve in the Eastern Vidarbha Landscape. Inset showing study area location in the map of India

The study was primarily conducted at two neighboring sites in the Eastern Vidarbha Tiger Landscape within the greater Central Indian Tiger Landscape. The Two sites were; the Tadoba Andhari Tiger Reserve (TATR) and Navegaon Nagzira Tiger Reserve (NNTR) (Figure 1). TATR (19.95428 E to 20.51695 E and 79.12749 N to 79.73494 N) has an area of 625 km^2^ core and 1,127.17 km^2^ buffer, and NNTR (20.86209 E to 21.44738 E and 79.69802 N to 80.39064 N) has 656 km^2^ of core and 1,241.24 km^2^ of buffer. According to the biogeographic classification of India, both the study sites are in the Deccan Plateau zone (Rodgers & Panwar, 1988) and are located at a distance of ~ 85 km from each other. The study sites experience subtropical climate with three distinct seasons‐ summer, monsoon, and winter. The Reserves receive southwest monsoons with rainfall (1100–1500 mm) persisting from June to September. The Forest type is Southern tropical dry deciduous (Champion & Seth, 1968). *Tectona grandis* is the dominant species followed by *Terminalia tomentosa* and *Lagerstroemia parviflora* in the study sites. The terrain type is mostly plain with shallow valleys and rounded hills. Tiger, leopard, and dhole form the major predatory guild in the study sites and chital (*Axis axis)*, sambar (*Rusa unicolor)*, nilgai (*Boselaphus tragocamelus)*, wild pig (*Sus scrofa*), gaur (*Bos gaurus)*, barking deer (*Muntiacus muntjac)* are the major prey species (Dhanwatey et al., 2013).

### 2.2 Field and analytical method: Dhole pack size variation across study sites

In field studies, individual identification of dhole packs and their members is a challenge owing to their uniform pelage pattern (Modi et al., 2019). To overcome this challenge, we generated information on the spatial distribution of dhole packs in the study sites using a systematic camera trapping design optimized for large carnivores (Karanth & Nichols, 1998). The effective study area of 1,103.767 km2 and 1,273.116 km2 in TATR and NNTR, respectively, was divided into sampling grids of 2 km^2^. 381 grids in TATR and 596 grids in NNTR were covered for this exercise and pair of camera traps were active in each grid for 25–30 days. Camera trap locations were chosen based on the presence of indirect signs like tracks and dhole scats to maximize dhole‐captures. This resulted in the identification of intensive use areas by dholes in the study sites based on number of captures at each camera location. Being territorial and social species these intensive use areas are mutually exclusive for each pack and the probability of capture decreases as you move away from the core area (Roffler Waite et al., 2019). After identification of dhole intensive use areas across the study sites, an effort was made to actively track dholes on foot and using a vehicle. We located dholes while resting, on kills, and while moving across their intensive use areas and video‐graphed (Canon Powershot SX 50) them for estimating number of individuals in each dhole pack. This was done on weekly basis for each identified pack for the duration of 5 months.

### 2.3 a) Field and Analytical method: Variation in prey composition and density across study sites

Line transect‐based distance sampling (Buckland et al., 2001) was used to estimate prey densities at the two sites. Line transects were laid in a stratified random framework to ensure spatial coverage of all vegetation types. All transects were 2 km in length. The survey effort of 950 km and 984 km was put in TATR and NNTR, respectively. Data for species, group size, and composition, GPS (global positioning system) location of every observation, bearing of the animal using a compass and angular sighting distance using laser range finders, were recorded whenever sightings were made online transects.

### 2.4 b) Estimation of ungulate species across study sites

The individual density of all the species was calculated using Distance program version 6.2 (Thomas et al., 2010). We first examined the data from both sites for each species separately. Following this, the species observations at distances beyond which sightings were almost none were dropped or observations were binned to achieve model fit. Akaike information criterion and goodness‐of‐fit (GOF‐p) tests were used to judge and the fit of the model. Based on the selected model, individual density ($\hat{\mathrm{Di}}$) and estimates of group density ($\hat{\mathrm{Dg}}$) were derived for each species.

### 2.5 a) Field and Analytical method: Variation in tiger density across study sites

Capture–recapture‐based camera trapping was done to estimate densities of carnivores following standard protocols (Karanth & Nichols, 1998). A grid size of 2 km^2^ was used for camera trap placement. Based on the sign survey, camera traps were stationed on both sides of the trails, at junctions and water sources to maximize photo captures (Karanth & Nichols, 1998). Distance between the camera stations was between 1 km to 1.5 km to ensure spatial coverage in the sampling area and capture probability of the study population (Pollock et al., 1990). A closing period of 30 days was kept to ensure demographic closure.

### 2.6 b) Density estimation of Tiger across study sites

To estimate tiger densities at the two sites, maximum likelihood SECR (Borchers & Efford, 2008) approach was applied using secr 4.3.0 R package (Efford, 2020). We obtained capture probability at the activity center (g0) and spatial scale of detection (σ) that elucidates how the capture rate decreases with the increasing distance from activity center. SECR model prediction is based on the habitat mask that denotes habitat of the study area and possible locations that can act as activity centers for the individuals of the study population (Efford, 2011; Young et al., 2019). We created habitat suitability mask for both the tiger reserves and areas not suitable for activity centers (Villages and water bodies near around villages) were removed for the analysis (Grey et al., 2013). Further, based on movement parameter, we analyzed models at buffers of 15 km and 20 km for NNTR; and 12 km, 15 km, and 20 km for TATR, to account for individuals staying outside the buffer region of the tiger reserves and to get stable density estimates and accuracy (Devens et al.,^2018^, ^2020^; Kalle et al., 2011). We compared four models (null model, g0, σ, and g0 + σ) and the best fit model was chosen based on the lowest Akaike Information Criterion.

### 2.7 a) Data sources for distribution‐wide assessment of dhole pack size

Through Google scholar, we searched for scientific literature on pack size of dholes, using the keywords “Cuon alpinus”, “Dhole”, “Average”, “Mean”, and “Pack size”. Our search resulted in 34 scientific assessments from 1973 to 2018 that had reported average pack size of dholes. These 34 assessments belonged to 24 unique protected areas across dhole ranging countries in South and Southeast Asia. 18 of these unique sites were also a part of the recently published dhole diet review (Srivathsa et al., 2020). Subsequently, following a snowball sampling approach (Handcock & Gile, 2011), we referred to the aforementioned 34 assessments to collate data on tiger density along with prey density (of the closest or same assessment year). To investigate the effect of patch contiguity, we considered size of the protected area and to address terrain type of the protected areas, we considered elevational heterogeneity and terrain ruggedness index of the respective sites. The digital elevation model (DEM) available for global coverage was obtained from NASA’s Shuttle Radar Topography Mission 90m (SRTM 90m). Using DEM, we derived the range of elevation and terrain ruggedness index using terrain function in raster package in R 3.5, for each protected area. For further analysis, we used the interquartile range (IQR) of elevation and terrain ruggedness for respective protected areas.

### 2.8 b) Analytical methods

We used generalized linear models to examine correlation of dhole pack size reported from 24 unique sites across the dhole distribution range. We used only those studies (*n* = 29) for which data on all the predictor variables were available, that is, tiger density and ungulate density, size of the protected area (PA), elevational heterogeneity, and terrain ruggedness of the PA. We scaled predictor variables (Size of PA, elevational heterogeneity and terrain ruggedness), further, we checked for correlation among all predictor variables and dropped the correlated ones (r > 0.6), prior to analysis. We dropped elevational heterogeneity as the predictor variables because of its high correlation with terrain ruggedness. After screening for normal distribution of response variable, we used "Gaussian" family for the analysis. We used average dhole pack size as the response variable and tiger density, ungulate density, area of PA, terrain ruggedness as predictor variables. We analyzed a total of ten additive and interactive models (Table 2, Figure 3). We compared models with predictor variables based on our hypothesis and compared them to the null model (Intercept only). Model fits were compared using Akaike's Information Criterion corrected (AIC_C_), and the effect of parameters was gauged based on the direction and statistical significance of corresponding β‐coefficients. We used "MuMIn" package for model selection and averaging. Model selection was based on the difference between AIC models, (ΔAIC < 2) and 95% cumulative weight criteria. Model averaging was carried out for parameters based on top model selection. All analyses were performed in program R (R Development Core Team, 2014).

## 3 RESULTS

### 3.1 Pack size variation

**FIGURE 2:**
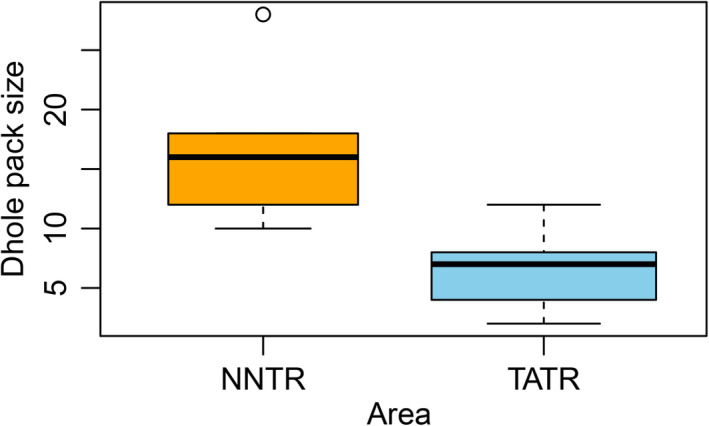
Comparison of dhole pack size from NNTR and TATR

We identified seven packs from TATR and five packs form NNTR. The number of individuals in a pack ranged from 7 to 12 for TATR Packs and 10 to 28 for NNTR Packs. The reported average pack size was 6.4 (1.3) and 16.8 (3.1) for TATR and NNTR, respectively. A significant difference was found between the pack size of TATR and NNTR (t = −3.05, p‐value = 0.02) as depicted through box plots (Figure 2, TATR pack size: median = 7, IQR = 4; NNTR pack size: median = 16, IQR = 6).

### 3.2 Prey composition and density across study sites

Total prey density per km^2^ was estimated to be 16.94 in NNTR and 19.28 in TATR. Major prey species in both the study sites were chital, sambar, nilgai, wild pig, gaur, and barking deer (Table 1). Gaur density was 5.21 (SE 1.41) the highest followed by chital 4.61(SE 1.2) in NNTR. In TATR, the density of chital was the highest 5.10 (SE 1.22) followed by sambar 4.68 (SE 0.76).

**TABLE 1:** Individual density of various prey species from NNTR and TATR, Maharashtra, India

Prey Species	NNTR (Individual density and SE)	Group size	TATR (Individual density and SE)	Group size
Chital	4.61 ± 1.2	5.02	5.10 ± 1.22	5.13
Sambar	1.41 ± 0.32	1.88	4.68 ± 0.76	2.25
Nilgai	1.99 ± 0.35	1.81	1.09 ± 0.36	2.50
Wild pig	3.12 ± 1.11	6.32	5.42 ± 2.08	7.22
Gaur	5.21 ± 1.41	5.98	2.03 ± 1.15	2.35
Barking deer	0.6 ± 0.2	1	0.96 ± 0.23	1.37

### 3.3 Large predator density across study sites

With the total sampling effort of 9,144 trap nights in TATR and 13,440 trap nights in NNTR, we obtained 452 and 211 photo captures on tigers in TATR and NNTR, respectively. A total of 58 tigers (Males = 32 and Female = 26) in TATR and 8 tigers (Males = 4 and Females = 4) in NNTR were identified through camera trap images. Based on Akaike information criterion, heterogeneity model (including baseline detection and movement parameter) (AICc 2,720.648) for TATR and null model for NNTR (AICc 739.167) and was chosen to be the best fit model. Tiger density estimate for TATR stabilized between 5.36 (SE 0.62) and 5.53 (SE 0.67) individuals per 100 km^2^ at 15 km and 20 km buffer. For NNTR estimated tiger density stabilized at 0.46 (SE 0.16) individuals per 100 km^2^ at 15 km buffer.

### 3.4 Distribution‐wide assessment of dhole pack size

**FIGURE 3:**
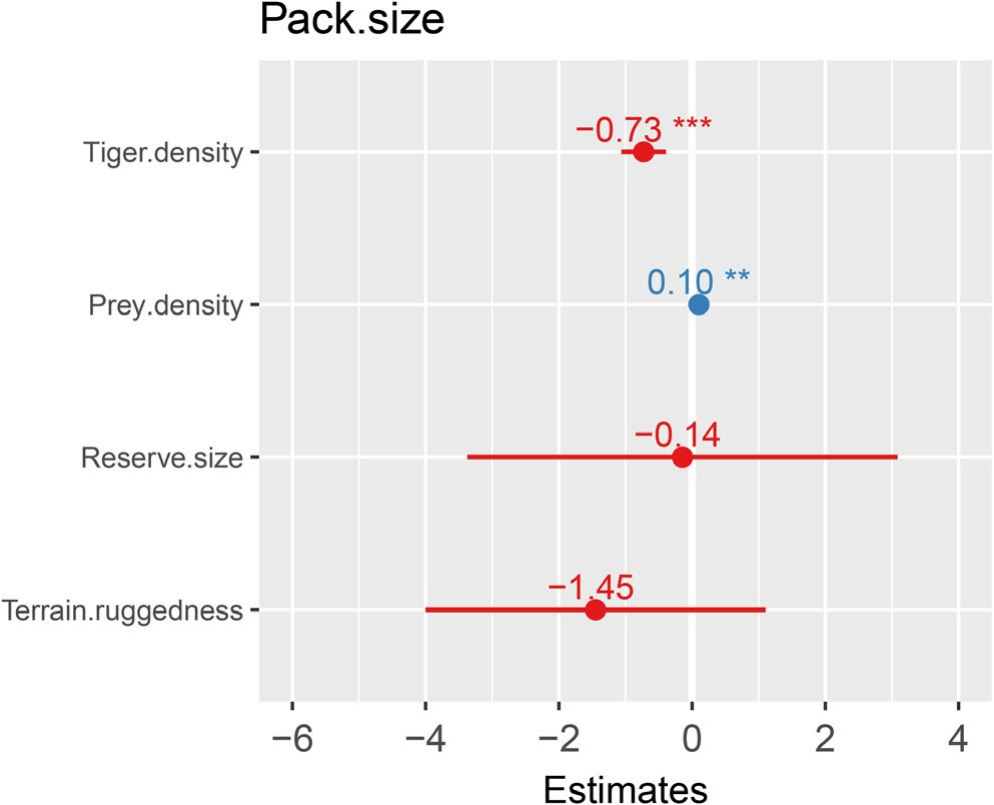
Showing the maximal model with all the significant and non‐significant predictor variables with β coefficient values at 95% confidence value

**FIGURE 4:**
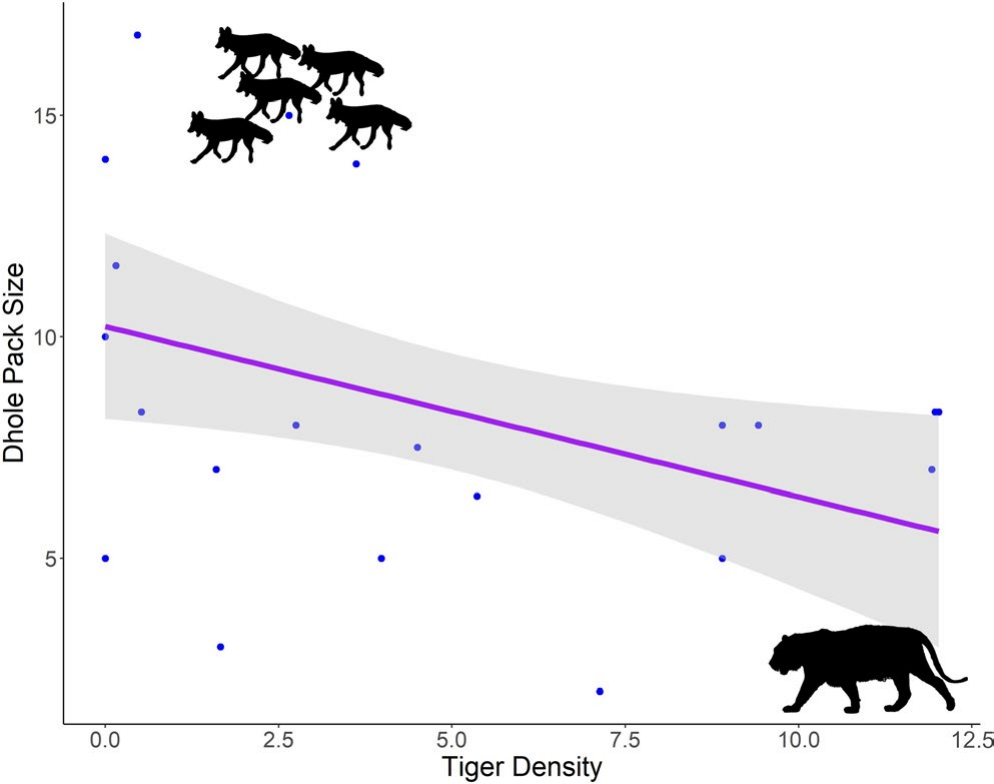
Dhole pack size in response to tiger density (per 100 km^2^) based on distribution‐wide assessment

**FIGURE 5:**
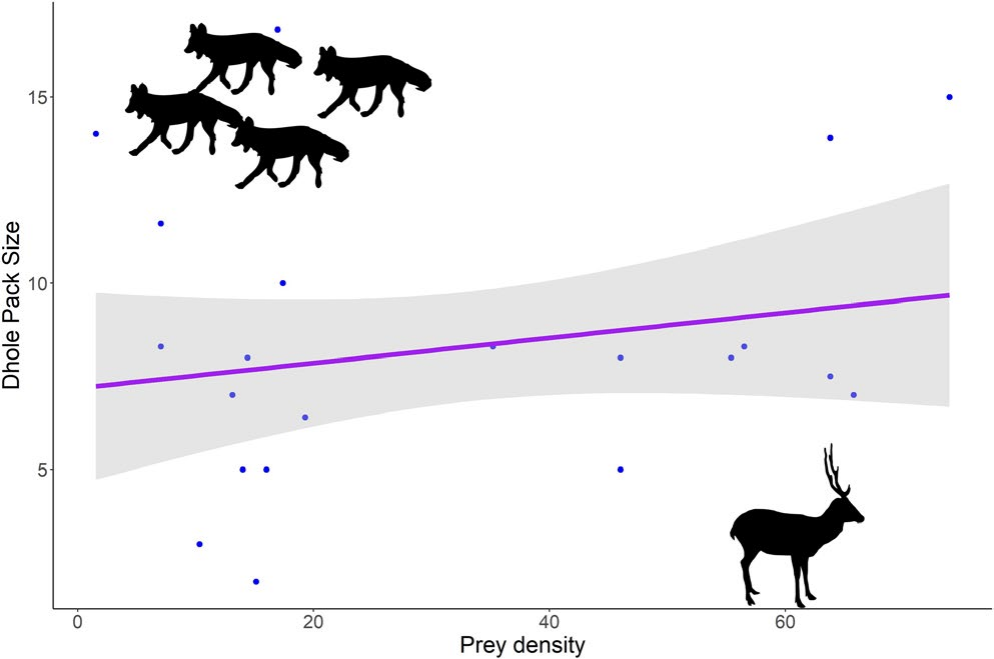
Dhole pack size in response to prey density (per km^2^) based on distribution‐wide assessment

**FIGURE 6:**
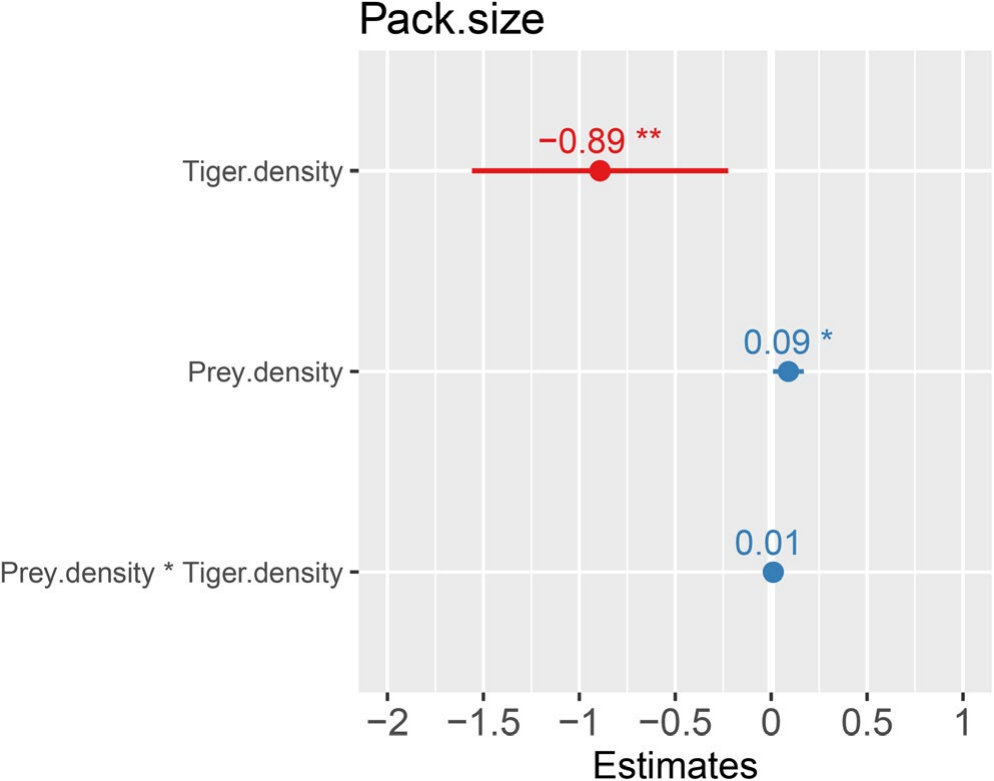
Average model parameters with β coefficient values at 95% confidence value

We used average dhole pack size as the response variable and tiger density, ungulate density, area of PA, terrain ruggedness as predictor variables (Figure 3). Out of the 10 additive and interactive models, top two models achieved the model selection criterion of ΔAICc < 2 and 95% cumulative weight criteria. Upon model selection we found, an additive effect of tiger density (effect size −0.77) and prey density (effect size 0.64) and interactive effect of tiger density (effect size −0.83) and prey density (effect size 0.73), tiger density* prey density (0.27), to be the top two best models (Table 2, Figures 4&5). Here, effect size values (On a scale of 0 to 1) show relation between dependent and independent variables, larger the value of effect size stronger is the influence of independent variable on the dependent variable. The Negative sign shows the direction of relation between the independent variable and dependent variable. On averaging the two top models (Table 3, Figure 6), we found a negative association of tiger density (−0.89 ± 0.33, *p* =.01) and a positive association of prey density (0.09 ± 0.03, *p* =.03) with the pack size and prey*tiger density (0.01 ± 0.0, *p* =.18) was not significant but still explained the relation with the response variable.

**TABLE 2:** Model comparison and selection following AIC_C,_ and 95% cumulative weight criteria

		Intercept	Prey.density	Reserve.size	Terrain.ruggedness	Tiger.density	Prey.density:Tiger.density	Terrain.rug	*df*	logLik	AICc	delta	weight	r^2^
**1**	**Tiger density + Prey density**	**8.43**	**0.11**	**NA**	**NA**	**−0.72**	**NA**	**NA**	**4**	**−71.40**	**152.48**	**0**	**0.562**	**0.44**
**2**	Tiger density * Prey density	**9.47**	**0.07**	**NA**	**NA**	**−1.15**	**0.01**	**NA**	**5**	**−70.36**	**153.32**	**0.85**	**0.368**	**0.48**
3	Tiger density + Prey density + PA size + Terrain ruggedness	11.26	0.10	−0.14	−1.449	−0.73	NA	NA	6	−70.67	157.16	4.69	0.054	0.47
4	Tiger density	10.24		NA	NA	−0.38	NA	NA	3	−77.27	161.50	9.03	0.006	0.16
5	Tiger density + Terrain ruggedness	13.18	NA	NA	−1.8474	−0.40	NA	NA	4	−76.45	162.57	10.10	0.004	0.21
6	Tiger density + PA size	16.09	NA	−1.91	NA	−0.46	NA	NA	4	−76.64	162.96	10.48	0.003	0.20
7	Null model	8.30	NA	NA	NA	NA	NA	NA	2	−79.93	164.32	11.84	0.002	0.00
8	Prey density	7.18	0.03	NA	NA	NA	NA	NA	3	−79.32	165.59	13.11	0.001	0.04
9	Prey density + Terrain ruggedness	9.43	0.03	NA	−1.3801	NA	NA	NA	4	−78.93	167.52	15.05	0.000	0.06
10	Prey density + PA size	3.60	0.04	1.13	NA	NA	NA	NA	4	−79.15	167.97	15.49	0.000	0.05
11	PA size + Terrain ruggedness	11.44	NA	−0.24	NA	NA	NA	−1.5847	4	−79.43	168.53	16.05	0.000	0.03

**TABLE 3:** Model averaging output for all variables present in the top model selection

	Estimate	Std. Error	Adjusted SE	z value	Pr(>|z|)	CI (2.5% ‐ 97.5%)
Intercept	8.839	1.229	1.280	6.906	<2e−16 ***	6.33 – 11.34
Tiger.density	−0.891	0.330	0.340	2.618	0.008*	−1.55 to −0.22
Prey.density	0.089	0.039	0.040	2.197	0.028 *	0.02 to 0.17
Prey.density*Tiger density	0.011	0.008	0.008	1.303	0.192	−0.01 to 0.028

Note

Signif. codes: 0 ‘***’ 0.001 ‘**’ 0.01 ‘*’ 0.05 ‘.’ 0.1 ‘ ’ 1

## 4 DISCUSSION

Across a wide range of taxa from oceanic (Baum & Worm, 2009) to terrestrial ecosystems, competitively inferior predators differ in abundance, distribution, and behavior as a response to apex predator density and distribution (Newsome et.al., 2017; Newsome & Ripple, 2015). Similarly, from the sympatric guild of tiger, leopard, and dhole, various scientific studies depict a significant decline in leopard populations along with the shift in their diet and spatial displacement to fringe areas, after the population recovery of tigers (Harihar et al., 2011; Kafley et al., 2019; Mondal et al., 2012; Steinmetz et al., 2013). Our study also revealed an inverse relationship between the density of tiger and group size of dhole while accounting for variability in resources and habitat heterogeneity.

Dhole pack size in NNTR was ~ 2.62 times bigger than the pack size in TATR. The smaller pack size of dholes in high tiger density scenario could be because of two reasons. Firstly, intense intraguild competition and associated risk of fatal injuries negatively affect dholes in TATR. For instance, loss of experienced breeders due to predation can result in decreased reproductive rate and destabilization in the pack; mortalities of helpers in the pack can result in diminished hunting efficiency and reduced food provisioning for pups; litter loss due to predation events from tiger can result in lower recruitment rates in the pack (Borg et al., 2015; Courchamp & Macdonald, 2001). All these mechanisms would synergistically act to reduce pack size, beyond which a small pack would experience inherent challenges of breeding and survival. Secondly, larger groups can successfully defend their kills and also consume the prey quickly, leaving minimal chances to attract other competitors (Carbone et al., 2005). However, the fact that per capita intake is compromised in larger groups because of sharing carcass with a greater number of individuals, might act as a counteractive selective pressure. Therefore, we predicted that the decision to be in smaller groups is to maximize energy gain while coexisting with dominant predator by showing differential prey selection in prey size class and to avoid detection over kills. Although interference competition events are difficult to observe in the wild in such a landscape, we collected opportunistic data via direct predation events, that is, tigers killing dholes (*n* = 5) and occasions when dholes lost their kills to tigers (*n* = 23), which support our predictions on the relation between the two competing species. Long‐term studies on the demography and behavioral ecology of the two species would further our understanding of such intraguild interactions in the landscape.

Conversely, low apex predator density in NNTR seems to be operating in a complex two‐way mechanism. We predicted that the larger pack size of dholes in NNTR might be an outcome of reduced predation pressure and easy availability of resources. It is observed in multipredator systems that availability of prey resources is key to the coexistence among carnivores. However, external factors like human‐mediated disturbances can alter trophic interactions. Declining apex predator population often results in the reduced threat of predation and wider niche availability in terms of food and space for subordinate predators (Green et al., 2019). Such competitive release scenarios lead to higher survival rates of juveniles and subadults which correspond to larger group sizes in subordinate carnivores (Groom et al., 2017). Additionally, larger packs of subordinate predator are also known to be competitively advantageous to suppress the recovery of dominant predator in the system (Periquet et al., 2015).

Patterns at local scale were also in concurrence with results of distribution‐wide assessment of dhole pack size. We found pack size to be negatively associated with tiger density and positively associated with prey density. However, the effect size of tiger density was stronger than that of prey density. A similar pattern has been observed in the African ecosystem, where lion populations crashed due to human‐induced environmental changes while facilitating the spotted hyena population. Spotted hyenas exhibit more behavioral plasticity than lions and have adapted to human subsidies in disturbed habitats. The reduced lion abundance and decreased risk of predator encounter resulted in increased foraging group size, larger clan size, an overall increase in time spent on kill sites, and easy availability of human subsidies for spotted hyenas (Green et al., 2019). Another such trend has been observed between African wild dog pack size and lion densities across temporal scale in Savé Valley Conservancy (SVC), Zimbabwe (Groom et al., 2017). Before lion population recovery, wild dog pack sizes were observed to be large. However, after the population recovery of the dominant predator, the wild dogs suffered a significant decline in survival rates of pups and adults.

Previous studies reported a positive correlation between group size and patch contiguity (Atwood, 2006) although, the current study failed to find this relationship. However, we suggest such factors can be tested at a fine‐scale to infer the effect of anthropogenic disturbances, protection level at reserve level, and connectivity in the landscape (Greenville et al., 2014; Newsome & Ripple, 2015). Interestingly, we did not find pack size to be correlated to terrain type; however, previously small pack size of dholes has been reported from areas with rugged terrain in Northeast India and Loas (Selvan et al., 2014; Kamler et al. 2020). This could be due to two reasons, small representation of areas with rugged terrain in comparison with flatter terrains and small pack size in rugged terrain might be a local adaptation and would not reflect at the global level.

Lastly, we suggest that a detailed understanding of the guild warrants serious consideration rather than species centric conservation approach. India is among the 13 countries that envision the goal of TX2 by 2022 for global recovery of wild tiger populations. According to the recent All India Tiger Estimation project, India harbors an estimated tiger population of 2,967 (Jhala et al., 2020). Despairingly, the global population of Asiatic wild dog/ dhole is roughly around 949–2215 mature individuals (Kamler, 2015). Within India, the persistence of dholes is mostly confined to protected areas with infrequent presence records from secondary forests and agro‐forest plantations (Srivathsa et al., 2019). These remnant habitat patches are also shared by other large carnivores, thereby limiting the dhole population by lethal intraguild interactions (Steinmetz et al., 2013). The small size of PAs and lopsided conservation efforts can further result in over‐inflated apex carnivore densities and detrimental for subordinate predators like dholes (Karanth et al., 2010; Kumar et al., 2019; Rayan & Linkie, 2016).

Competition from dominant carnivores remains one of the major factors that drive densities of other sympatric carnivores in a system. Therefore, to conserve the endangered dhole, it is crucial to understand its response to tigers, another endangered and flagship species. Our study fills some glaring lacunae in the previous understanding of how intraguild competition can potentially limit a subordinate social predator. NNTR and TATR were comparable natural experiment setup that allowed us to understand demographic responses and the consequences of dominance shift between the endangered tropical carnivores. We suggest that the increased pack size of a social subordinate predator seems to be a demographic adaptation to varying competitor densities and availability of wider niche. This study contributes to the holistic understanding of guild interactions to optimize ecological triage while conserving charismatic apex predators and mid‐ranking predators.

## CONFLICT OF INTERESTS

Authors declare they have no competing interests.

## AUTHOR CONTRIBUTION

**Aishwarya Bhandari:** Conceptualization (equal); Data curation (equal); Formal analysis (lead); Methodology (equal); Validation (equal); Writing‐original draft (lead); Writing‐review & editing (equal). **Pallavi Ghaskadbi:** Conceptualization (equal); Data curation (equal); Formal analysis (equal); Methodology (equal); Validation (equal); Visualization (equal); Writing‐original draft (equal); Writing‐review & editing (equal). **Parag Nigam:** Conceptualization (equal); Data curation (equal); Supervision (equal); Validation (equal); Writing‐review & editing (equal). **Bilal Habib:** Conceptualization (lead); Data curation (equal); Formal analysis (equal); Funding acquisition (lead); Investigation (lead); Methodology (equal); Project administration (lead); Resources (lead); Supervision (lead); Validation (equal); Visualization (equal); Writing‐review & editing (equal).

## 5 ETHICS STATEMENT

This study was carried out under the research permit number D‐22(8)/WL/Research/CT‐722/(12–13)/2934/2013 from Maharashtra Forest Department. During the study, only noninvasive methods were used for the sampling purpose therefore animal handling permits were not required.

## AUTHORS’ CONTRIBUTION

B.H., P.N., A.B., and P.G.: Conceiving ideas. B.H., A.B., and P.G.: study design. A.B. and P.G.: Data collection; data analysis. A.B. and B.H.: Manuscript reading and leading. B.H., P.N., A.B., and P.G.: Approval of final draft.

## Data Availability

All data are submitted along with manuscript in the form of supplementary material.
